# A method to develop vocabulary checklists in new languages and their validity to assess early language development

**DOI:** 10.1186/s41043-018-0145-1

**Published:** 2018-05-11

**Authors:** Elizabeth L. Prado, John Phuka, Eugenia Ocansey, Kenneth Maleta, Per Ashorn, Ulla Ashorn, Seth Adu-Afarwuah, Brietta M. Oaks, Anna Lartey, Kathryn G. Dewey

**Affiliations:** 1 Department of Nutrition, University of California Davis, 3253 Meyer Hall, One Shields Ave, Davis, CA 95616 USA; 2 School of Public Health and Family Medicine, University of Malawi College of Medicine, Private Bag 360, Chichiri, Blantyre 3, Malawi; 3 Department of Nutrition and Food Science, University of Ghana, Legon, Box LG 134, Legon, Accra, Ghana; 4 Center for Child Health Research, University of Tampere Faculty of Medicine and Life Sciences and Tampere University Hospital, Arvo building, FIN 33014 Tampere, Finland; 5 Department of Paediatrics, Tampere University Hospital, POB 2000, FIN 33521 Tampere, Finland; 6 Department of Nutrition and Food Sciences, University of Rhode Island, 131 Fogarty Hall, Kingston, RI 02881 USA

**Keywords:** Developmental assessment, Predictive validity, Concurrent validity, Low- and middle-income countries, Cross-cultural assessment

## Abstract

**Background:**

Since the adoption of United Nations’ Sustainable Goal 4.2 to ensure that all children have access to quality early child development (ECD) so that they are ready for primary education, the demand for valid ECD assessments has increased in contexts where they do not yet exist. The development of early language ability is important for school readiness. Our objective was to evaluate the validity of a method to develop vocabulary checklists in new languages to assess early language development, based on the MacArthur-Bates Communicative Development Inventories.

**Methods:**

Through asking mothers of young children what words their children say and through pilot testing, we developed 100-word vocabulary checklists in multilingual contexts in Malawi and Ghana. In Malawi, we evaluated the validity of the vocabulary checklist among 29 children age 17–25 months compared to three language measures assessed concurrently: Developmental Milestones Checklist-II (DMC-II) language scale, Malawi Developmental Assessment Tool (MDAT) language scale, and the number of different words (NDW) in 30-min recordings of spontaneous speech. In Ghana, we assessed the predictive validity of the vocabulary checklist at age 18 months to forecast language, pre-academic, and other skills at age 4–6 years among 869 children. We also compared the predictive validity of the vocabulary checklist scores to that of other developmental assessments administered at age 18 months.

**Results:**

In Malawi, the Spearman’s correlation of the vocabulary checklist score with DMC-II language was 0.46 (*p* = 0.049), with MDAT language was 0.66 (*p* = 0.016) and with NDW was 0.50 (*p* = 0.033). In Ghana, the 18-month vocabulary checklist score showed the strongest (rho = 0.12–0.26) and most consistent (8/12) associations with preschool scores, compared to the other 18-month assessments. The largest coefficients were the correlations of the 18-month vocabulary score with the preschool cognitive factor score (rho = 0.26), language score (0.25), and pre-academic score (0.24).

**Conclusions:**

We have demonstrated the validity of a method to develop vocabulary checklists in new languages, which can be used in multilingual contexts, using a feasible adaptation process requiring about 2 weeks. This is a promising method to assess early language development, which is associated with later preschool language, cognitive, and pre-academic skills.

**Electronic supplementary material:**

The online version of this article (10.1186/s41043-018-0145-1) contains supplementary material, which is available to authorized users.

## Background

The post-2015 sustainable development goals have placed early child development (ECD) on the global policy agenda for the first time, as the United Nations’ 193 members have adopted goal 4.2 to “ensure that all girls and boys have access to quality early childhood development, care and pre-primary education so that they are ready for primary education.” The adoption of this goal has created an increasing demand for standard ECD assessment methods in low- and middle-income countries, where such assessments commonly do not yet exist. ECD assessments are needed to track progress toward this goal, to screen children for further evaluation and diagnosis, and to evaluate programs and interventions to inform evidence-based policy.

Early language development is especially important for school readiness. While some children who begin talking later than their peers catch up in vocabulary a few months later, others continue to lag behind their peers and remain at risk for language disorders [1]. Early measures of language ability predict later IQ, reading, and math achievement at school-age [2, 3]. Assessing language development can be challenging in low- and middle-income countries, where it is common for multiple languages to be spoken. Standardized language assessment tools do not usually exist in the local languages, and the development of such tools can consume a substantial amount of time and resources. Early language assessments are needed that can be easily developed for new languages and are appropriate in multilingual contexts.

Where standard ECD tests do not exist, assessment methods from another language and context are commonly adopted or adapted, or a new test is assembled [4]. Adoption, adaptation, and assembly are not delineated categories, but represent a spectrum of adaptation procedures. At the adoption end of the spectrum, a test is directly translated to a new language and context without modification. However, test items, materials, and procedures are often inappropriate for children in a new context and must be adapted [5]. More extensive modifications or merging items from multiple sources lead to the assembly of a new test. Few studies have reported evidence for the validity of ECD tests that have been adopted, adapted, or assembled in low- and middle-income country contexts. A review of 114 publications reporting the use of ECD assessments in low- and middle-income countries found that many of the studies did not report any information on validity [6]. The objective of the current study was to evaluate the validity of a method to develop vocabulary checklists in new languages to assess early language development, based on the MacArthur-Bates Communicative Development Inventories (CDI).

## Methods

This study was conducted as a part of the International Lipid-Based Nutrient Supplements (iLiNS) Project in Ghana and Malawi. In the iLiNS-DYAD-G trial in Ghana (*n* = 1320) and the iLiNS-DYAD-M trial in Malawi (*n* = 869), pregnant women were enrolled before 20 weeks of gestation. In the iLiNS-DOSE trial in Malawi (*n* = 1932) infants were enrolled at age 6 months. All participants were assigned to receive various doses and formulations of lipid-based nutrient supplements, or to control groups until age 18 months, when child development was assessed [7–9]. The effects of the interventions on 18-month vocabulary and other developmental scores, which were not significant in any trial, have been reported previously [10–12].

In the current study, we evaluated the validity of the vocabulary checklists developed for the iLiNS trials. In Malawi, we evaluated the validity of the vocabulary checklist scores in comparison to three other language assessments measured concurrently: the Developmental Milestones Checklist-II (DMC-II) language scale administered by caregiver interview, the Malawi Developmental Assessment Tool (MDAT) language scale, administered by direct child assessment, and the number of different words spoken by the child in naturalistic speech samples. In Ghana, we evaluated the predictive validity of the vocabulary checklist scores at age 18 months to forecast language, pre-academic, and other skills at age 4–6 years. We also compared the predictive validity of the vocabulary checklist scores to that of other developmental assessments administered at age 18 months.

Ethical approval for the study procedures was obtained from the Institutional Review Board of the University of California Davis or the Ethics Committee at Pirkanmaa Hospital District, Finland, as well as the University of Malawi, College of Medicine Research and Ethics Committee or the Ghana Health Service and the University of Ghana Noguchi Memorial Institute for Medical Research. All participants provided written informed consent, by signature or thumb-print of a parent on behalf of the children. Children’s assent was indicated by their willingness to participate in the activities.

In Ghana, the study area was semi-urban and maternal education averaged 8 years in the study sample. In Malawi, the study area was partly rural and partly semi-urban and maternal education was 4 years, on average. Children in both contexts experienced linear growth faltering, with length-for-age *z*-score at age 18 months below the mean of World Health Organization norms [13] in Ghana, on average 0.8 SD below the mean, and in Malawi, on average 1.8 SD below the mean.

### Participants and procedures: concurrent validity of vocabulary scores in Malawi

To assess the concurrent validity of the language assessments, we enrolled 30 children age 17–25 months (mean 20.8, SD 2.1) who resided in the iLiNS-DOSE study area but did not participate in any iLiNS trial. The iLiNS-DOSE trial was conducted in two catchment areas served by the Mangochi District Hospital and the Namwera Health Centre. We divided the Mangochi area into four quadrants and selected one village in each quadrant from which to recruit participants. We divided the Namwera area into two halves and selected one village from each half. In these six villages, project staff obtained lists of children within the target age range from community health workers. They visited the homes of these children to recruit participants until they reached the target sample size of five children per village. We powered the study to detect a correlation of 0.50, which would indicate moderate concurrent validity. A sample size of 30 provides 80% power to detect that a Spearman’s correlation of 0.5 is greater than zero with an alpha of 0.05 in a two-sided test.

After obtaining informed consent, project staff administered the DMC-II language scale at this home visit and scheduled a clinic visit for the following week. At the clinic visit, the vocabulary checklist and the MDAT language scale were administered. Within 2 weeks of enrollment, project staff visited the participant’s home to video and audio-record the child for 3–4 h in his or her natural environment. Children wore a small backpack containing a high-quality digital recorder (Zoom H2 Ultra-Portable Digital Audio Recorder) connected to a lapel microphone attached to the child’s shirt near his or her mouth. We instructed the caregivers and children to carry on their normal daily activities while the videographer recorded from a distance to intrude as little as possible.

Two transcribers were trained on the Codes for the Human Analysis of Transcripts (CHAT) transcription system [14]. For each transcript, a transcriber listened to the entire recording, then transcribed a 30-min segment in which the child was talkative. A supervisor checked a randomly selected 5-min segment of each transcript against the recording and counted the number of words in each utterance and the number of errors. Average accuracy across transcripts was 97%. We computed each child’s number of different words (NDW) spoken during the 30-min transcript using Computerized Language Analysis (CLAN) software.

### Participants and procedure: predictive validity of 18-month developmental assessments in Ghana

We evaluated the predictive validity of the iLiNS 18-month developmental assessments using data from the iLiNS-DYAD-G trial in Ghana. In 2011–2014, all trial participants were invited to a clinic visit for developmental assessment at age 18 months, including the vocabulary checklist, Kilifi Developmental Inventory, Profile of Socio-Emotional Development, A not B task, and family care indicators interview. These assessments were completed for 1023 children (mean 18.2, SD 0.3 months). In 2016, we re-enrolled 966 children in a follow-up study, 869/1023 (85%) of whom had been assessed at age 18 months. We assessed their motor, cognitive, and socioemotional development at a clinic visit at preschool age (mean 4.9, SD 0.5 years).

### Method to develop vocabulary checklists

In Malawi and Ghana, we developed 100-word vocabulary checklists in the local languages based on the MacArthur-Bates CDI [15], in part following previous adaptations of this tool in Bangladesh [3] and Kenya [16]. The local languages in the project areas were Chichewa and Chiyao in Malawi, and in Ghana, they were Krobo, Ewe, Twi, and English. Project staff conducted interviews with 41 mothers of children age 14 to 33 months in Malawi and 23 mothers of children age 14 to 27 months in Ghana, asking mothers what words their children said, and probing specific categories from the MacArthur-Bates CDI, such as animals, food, and clothing. We used the results of these interviews to develop a list of 352 words in Malawi and 240 words in Ghana. We then asked 41 additional mothers of children age 13 to 23 months in Malawi and 19 additional mothers of children age 12 to 31 months in Ghana whether their children said each of these words. For each word, the child was given credit for saying that word in any language.

Using these data, we selected 100 words with a range of item difficulty (easy, moderate, and advanced). In Malawi, to select words in the “easy” category, we selected all 18 words for which 50–100% of respondents answered positively. For words in the “moderate” (30–50% responded positively) and “advanced” (10–30% responded positively) groups, we only considered words with a positive correlation with age and positive correlation with total vocabulary. From the words that met these criteria, we selected a representative sample of words from each category (e.g., food, household objects, animals). In Ghana, we used slightly different cutoffs for easy (70–100% responded positively), medium (50–70% responded positively), and advanced (20–50% responded positively) compared to Malawi because the children who participated in the pilot study in Ghana were slightly older (mean age 23 months in Ghana versus mean age 18 months in Malawi). For each group of words (easy, medium, and advanced), we selected a representative sample of words from each category (e.g., food, household objects, animals) which had a positive correlation with age and total vocabulary score. In each country, this method to develop the vocabulary checklists required about 2 weeks.

### Other 18-month language assessments

The MDAT was assembled in Malawi, originally from items selected from the Denver Developmental Screening Tool, Denver-II, and Griffiths Mental Development Scales [17]. We administered the 34-item MDAT language scale mainly by child observation, though five items can be reported by the caregiver if the child refuses to perform the skill (e.g., “can sing songs or repeat rhymes from memory”). The score was the number of language items the child was able to perform [17]. The MDAT was previously validated in Malawi. More than 94% of items showed high reliability (kappa > 0.4 for inter-observer immediate, delayed, and intra-observer reliability) [17]. Using the screening criterion defined as whether the child failed two items or more in any one domain at the chronological age at which 90% of the normal reference population would be expected to pass, the MDAT demonstrated high sensitivity (97%) and specificity (82%) to detect children with neurodevelopmental impairment in Malawi [17].

The DMC was assembled in Kenya by adapting items selected mainly from the Griffiths Mental Development Scales and Vineland Adaptive Behavior Scale [18]. The first version of the DMC was further adapted and extended for the iLiNS-ZINC trial in Burkina Faso, creating the DMC-II [19]. The DMC-II scores demonstrated internal reliability (Cronbach’s alpha), inter-interviewer, and test-retest reliability (intraclass correlation coefficient) of greater than 0.75 and showed expected correlations with age, stunting, wasting, and underweight in Burkina Faso [19]. We administered the 16-item DMC-II language scale in Malawi by caregiver interview and calculated the score as the sum of the item scores.

### Other 18-month assessments

The KDI motor assessment was also assembled in Kenya drawing motor items from several standard tests, including the Griffiths Mental Development Scales and the Merrill-Palmer Scales [20]. Using the 10th centile as a cutoff, the KDI showed 89% sensitivity and 91% specificity to detect children with neurodevelopmental impairment in Kenya [20]. The child’s score was the number of items he or she was observed to perform out of 34 fine motor skills, for example “threads two beads onto shoe lace” and 35 gross motor skills, for example “walks on tip toes for three or more steps.”

The Profile of Socioemotional Development (PSED) was developed in Kenya based on the Child Behavior Questionnaire for Parental Report [21], with additional items from the Brief Infant/Toddler Social Emotional Assessment (BITSEA) (Abubakar A, Holding P, Mwangome M, Kabunda B, Kalu R, Maitland K, Newton C, Van de Vijver FJR: The profile of social and emotional development, a conversational approach to the systematic monitoring of children’s social and emotional development, unpublished). The PSED was designed as a structured interview to elicit from a caregiver descriptions of the child’s daily behavior, which were used to code 19 items on a scale from 0 to 2 [21]. Excluding two items that did not correlate with the total, *Cronbach’s Alpha*, indicating internal reliability, was 0.75 among 2000 children in Malawi and 0.67 among 1022 children in Ghana. These 17 items were summed for a total score, which indicated higher socioemotional problems. Since other standard socioemotional assessments, such as the BITSEA and Strengths and Difficulties Questionnaire calculate separate scores for socioemotional competence and problems, we also calculated a social competence score (7 items) and a behavioral problem score (10 items). We classified PSED items as competence or problem items based on the BITSEA classification, because most of the PSED items overlapped with BITSEA items.

The A not B task is a widely used test of working memory and executive function in young children that has been previously adapted in Kenya [22, 23]. In each of 10 trials, a small snack was hidden under one of two identical cups on a board. After a delay of 5 sec, the child was invited to find the snack. Every time the child achieved two correct consecutive trials, the snack was hidden at the alternate location. The scores were the total correct trials and perseverative errors (the total number of errors committed after the first set of two correctly solved trials).

We assessed the child’s home environment at age 18 months with the family care indicators (FCI) interview [24]. For each of six activities (e.g., told stories, sang songs), we asked the caregiver (98% mothers) whether the child’s mother, father, and any other adult had engaged in that activity with the child in the past 3 days. We also asked 12 additional questions concerning toys and books in the home. We calculated three scores: (1) the total FCI score as the sum of all 18 items representing 6 activities plus 12 additional items, (2) the variety of play materials as the sum of 7 items concerning toys in the home, and (3) activities with caregivers as the sum of the 18 item scores representing 6 activities for each of the three categories of potential caregivers.

### Preschool assessments

Table 1 describes the tests we used to assess preschool cognitive, motor, and socioemotional development in Ghana. For further details, see Additional file 1. We assessed nurturing and stimulation at preschool age with the Early Childhood version of the Home Observation for the Measurement of the Environment (HOME) Inventory [25], which we adapted to the local context through focus groups and pilot testing.

**Table 1:** Preschool developmental assessment methods in Ghana

Preschool score	Method
Cognitive	
Pre-academic skills	Parents’ evaluation of developmental status: developmental milestones pre-academic subscale.
Paired-associate memory	The child was taught novel names for eight objects and tested for recall and recognition.
Language	Developmental Neuropsychological Assessment (NEPSY-II) body part naming and identification and comprehension of instruction sub-tests.
Block design	Adapted British Abilities Scales pattern construction sub-test.
Visual search	Adapted NEPSY visual search sub-test.
Inhibitory control: head/toe task	Head/toe sub-test of the International Development and Early Learning Assessment (IDELA).
Inhibitory control: delay of gratification	Whether the child chose to receive one treat immediately or to wait for two, three, or four treats at the end of the second, third, and fourth tasks, respectively.
Cognitive factor score	Factor score calculated as the first component of a factor analysis using principal-axis factoring method including all cognitive *z*-scores except delay of gratification, which was the only score that was not strongly associated with the others. This component accounted for 79% of the variance in these scores.
Motor	
Fine motor	National Institute of Health (NIH) Toolbox 9-Hole Pegboard test.
Socioemotional	
Socioemotional Competence and Difficulties	Strengths and Difficulties Questionnaire (SDQ)
Behavior rating scale	Adapted from the Preschool Self-Regulation Assessment (PSRA) Assessor Report.

### Training and personnel

In Malawi, 15 data collectors and, in Ghana, 6 data collectors were trained to administer the CDI, KDI, PSED, and A not B task for the iLiNS 18-month developmental assessments. In Ghana, 5 data collectors were trained to administer the preschool assessments. The educational background of the data collectors ranged from a high school degree to a 4-year post-high school degree, and none had previous experience in developmental assessment. For the 18-month assessments, after 1 month of training, including practice, coaching, and feedback, all data collectors reached proficiency in administering the tests, demonstrated by high scores (> 80%) on written tests, practical evaluations, and inter-rater agreement, as previously reported [10–12]. Inter-rater accuracy of each data collector compared to her supervisor was also high (> 90%) on all of the preschool tests, except visual search (74%), due to slight differences between data collectors and the supervisor in regulating stopwatches (mean difference 2.4 s). For the language validation study, two of the developmental assessment staff in Malawi were trained to administer the DMC-II language and MDAT language scales.

### Statistical analysis

Missing item data occurred on the caregiver-report tools if the caregiver did not know the response and on the direct assessments if the child refused to attempt to perform the activity. The percentage of missing item scores was low for the caregiver-report tools (< 0.5% of item scores for the CDI, DMC-II, and PSED) and higher for the tools administered by child observation (MDAT 9%, KDI 9%, A not B 5%). For the MDAT and KDI, we performed single imputation of missing item scores using the method described in Raghunathan et al. [26] before calculating total scores. In this method, the imputation is performed by fitting a sequence of regression models and drawing values from the corresponding predictive distributions. By this method, we used the available item scores to predict the missing items. For the other tests, we considered missing item scores to be a failure, since there was only a very small percentage of item scores missing and in cases where the caregiver did not know or the child refused, it was likely that the child was not able to perform the skill.

We evaluated concurrent validity of the language scores using Spearman’s correlations. We evaluated predictive validity by computing Spearman’s correlations between each 18-month score and each preschool *z*-score, calculated by 3-month age bands. We used Spearman’s rank correlations because not all scores were normally distributed. Spearman’s method does not assume a normal distribution and is robust to outliers. All *p* values were corrected for multiple hypothesis testing using the Benjamini-Hochberg method [27]. All analyses were conducted using SAS version 9.4 (SAS Institute, Cary, NC).

## Results

### Concurrent validity in Malawi

Of the 30 children enrolled in the language validation study, one dropped out after language assessment but before audio recording. The number of child utterances in the 30-min speech samples ranged from 2 to 304 (mean 118, SD 80). The Spearman’s correlation (*n* = 29) of NDW with CDI vocabulary was 0.50 (*p* = 0.033), with MDAT language was 0.23 (*p* = 0.378) and with DMC-II language was 0.47 (*p* = 0.050). Due to the wide variance in the number of child utterances in the speech recordings, we performed an additional analysis excluding three children with less than 20 utterances, in which case it is likely that the speech sample was not representative of the child’s vocabulary. Excluding these children, these correlations increased to 0.58, 0.35, and 0.53, respectively (Table 2). Excluding two additional children with > 10% missing MDAT items, the correlation of MDAT language score with NDW increased to 0.39. The correlation (*n* = 30) of CDI with MDAT language was 0.66 (*p* = 0.016), of CDI with DMC-II language was 0.64 (*p* = 0.007), and of MDAT with DMC-II language was 0.46 (*p* = 0.049). Excluding four children with > 10% missing MDAT items, these correlations increased to 0.72 with CDI and 0.50 with DMC-II.

**Table 2:** Concurrent Validity of Several Measures of Early Language Development in Children age 17–25 Months in Malawi

Assessment 1		Assessment 2			
Score 1	Method 1	Score 2	Method 2	*n*	rho^2^
CDI vocabulary score	Caregiver interview	NDW^1^	Naturalistic observation	26	0.58*
MDAT language score	Child directly tested	NDW^1^	Naturalistic observation	26	0.35
DMC-II language score	Caregiver interview	NDW^1^	Naturalistic observation	26	0.53*
CDI vocabulary score	Caregiver interview	MDAT language score	Child directly tested	30	0.66**
CDI vocabulary score	Caregiver interview	DMC-II language score	Caregiver interview	30	0.64**
DMC-II language score	Caregiver interview	MDAT language score	Child directly tested	30	0.46*

All *p* values were corrected for multiple hypothesis testing using the Benjamini-Hochberg correction

^1^Number of different words in a 30-min speech sample, excluding one child who dropped out and three children with less than 20 utterances

^2^Spearman’s correlation

*p < 0.05

**p < 0.01

### Predictive validity in Ghana

The sample of 869 children included here did not differ significantly from the 451 who were enrolled but not included in demographic characteristics such as maternal education and household asset index. The Spearman’s correlations of the 18-month scores with cognitive, motor, and socioemotional scores at preschool age are presented in Table 3. Of the 18-month scores, CDI vocabulary showed the strongest and most consistent associations with preschool scores, significantly correlated with 8/12 preschool scores, followed by the FCI total score and variety of play materials, each of which was significantly correlated with 4/12 preschool scores. The largest coefficients were the correlations of the CDI with the cognitive factor score (rho = 0.26), language score (0.25), and pre-academic score (0.24). Children with higher 18-month vocabulary scores had higher scores in inhibitory control (0.16) and paired-associate memory (0.16), and faster visual search (− 0.12) and fine motor speed (− 0.13) at preschool age. However, children with higher 18-month vocabulary had significantly lower scores on the observed behavior rating scale, indicating poorer behavior during the preschool assessment (− 0.17).

**Table 3:** Predictive validity of 18-month ECD assessments in Ghana

	18-month domain:	Language	Motor			Executive function		Socioemotional			Home environment		
	18-month score:	CDI vocabulary checklist	KDI fine motor	KDI gross motor	KDI total motor	A not B total correct	A not B perseverative errors^1^	PSED competence	PSED problems^1^	PSED total^1^	FCI variety of play materials	FCI activities with caregivers	FCI total score
Preschool domain:	Preschool score:												
Cognitive	Cognitive factor score	0.26**	0.09^†^	–^2^	–	–	–	–	–	–	0.13**	0.08^†^	0.11**
Cognitive	Pre-academic score	0.24**	0.08^†^	–	0.09^*^	–	–	–	–	–	0.16**	0.18**	0.17**
Cognitive	Paired-associate memory score	0.16**	–	–	–	–	–	–	–	–	–	–	–
Cognitive	Block design score	–	–	–	–	–	–	–	–	–	0.14**	–	0.14**
Language	Language score	0.25**	0.08^†^	–	–	–	–	–	–	–	0.07^†^	–	–
Executive Function	Visual search speed^1^	− 0.12**	− 0.11**	− 0.09^†^	− 0.13**	–	–	–	–	–	− 0.13**	− 0.12**	− 0.13*^*^
Executive function	Inhibitory control: head/toe	0.16**	–	–	–	–	–	–	–	–	–	–	–
Executive function	Inhibitory control: delay of gratification	–	–	–	–	–	–	–	–	–	–	–	–
Fine motor	Pegboard fine motor speed^1^	− 0.13^**^	− 0.08^†^	–	− 0.09^†^	–	–	–	–	–	− 0.08^†^	–	− 0.08^†^
Socioemotional	SDQ total difficulties^1^	–	–	–	− 0.08^†^	–	–	− 0.08^†^	0.10^*^	0.10^*^	–	–	–
Socioemotional	SDQ prosocial	–	–	–	–	–	–	–	–	–	–	0.08^†^	–
Socioemotional	Behavior rating scale	− 0.17^**^	–	–	–	–	–	–	–	–	− 0.08^†^	–	− 0.08^†^

Ns for individual correlations range from 779 to 869. All *p* values were corrected for multiple hypothesis testing using the Benjamini-Hochberg correction

^1^A lower score represents better performance

^2^A dash (–) represents a correlation that was not significant or a trend (*p* > 0.1)

†*p* < 0.1

*p < 0.05

**p < 0.01

The KDI total motor score at 18 months was significantly correlated with preschool visual search speed and pre-academic skills (Table 3). The gross motor score was not significantly associated with any preschool scores, while the fine motor score was associated with visual search speed. The A not B total correct score and number of perseverative errors at 18 months were not significantly associated with any preschool scores. PSED total and problem scores at 18 months were significantly correlated with Strengths and Difficulties Questionnaire (SDQ) total difficulties, but were not associated with SDQ prosocial, observed behavior at preschool assessment, or any other scores.

For the FCI variety of play materials, activities with caregivers, and total FCI scores at age 18 months, the strongest correlations were found with preschool pre-academic skills (0.16–0.18), cognitive factor score (0.08–0.13), block design (0.14), and visual search speed (− 0.12–0.13). All three 18-month FCI scores were significantly associated with HOME Inventory score at preschool age: variety of play materials (rho = 0.21, *p* = 0.001), activities with caregivers (rho = 0.16, *p* = 0.001), and FCI total score (rho = 0.22, *p* = 0.001).

## Discussion

In both Malawi and Ghana, developing 100-word vocabulary checklists using the method we describe resulted in a practical and valid measure of early language development. Of the three language assessments conducted in Malawi (vocabulary checklist, DMC-II, and MDAT), the vocabulary checklist showed the highest correlation with concurrently measured NDW in spontaneous speech samples. The vocabulary checklist score also showed strong correlations with both DMC-II and MDAT language scores measured concurrently. Of the four 18-month developmental tests evaluated for predictive validity in Ghana (vocabulary checklist, KDI, A not B task, and PSED), the vocabulary checklist showed the strongest and most consistent associations with preschool cognitive scores.

The concurrent correlations that we found were similar in magnitude to those that have been found in previous studies of the CDI. In a study among children age 24 months in the USA, the 100-word CDI short form score was correlated with NDW in observations of mother-child semi-structured free play in the home (0.49) and with a language assessment administered by child observation (0.54) [28]. These are similar to our adapted CDI in Malawi, which correlated 0.58 with NDW and 0.66 with MDAT language. These findings show that adapting the CDI to a new context using the method we describe resulted in a tool that was comparable in validity to the tool in its original context.

A study in Colombia examined the validity of five parent-report tools compared to the Bayley Scales of Infant Development (BSID) administered by direct child assessment [29] among a sample of 1311 children age 6–42 months. In that study, standard tests originating from high-income countries were mainly adopted, with some adaptation of item wording and pictures. The concurrent validity of the vocabulary checklist in Malawi versus the observed MDAT score in our study (0.66) was much higher than that of any of the parent-report language tools evaluated in Colombia against the BSID language score at age 6–18 months (0.1–0.3) and slightly higher than the correlation of these tools with the BSID language score at 19–30 months (0.4–0.6) [29].

The magnitude of the predictive correlations of the CDI in our study is also comparable to results of previous studies. Analyses of the CDI expressive vocabulary short form at age 2 years predicting language scores at age 4–6 years in the USA and New Zealand have shown correlations of 0.35–0.45 [30, 31]. In Bangladesh, scores on a 60-word expressive vocabulary checklist at age 18 months showed correlations of 0.30–0.37 with WPPSI verbal, performance, and full scale IQ at age 5 years [3]. These are slightly higher than our findings in Ghana of correlations of 0.24–0.26 of the CDI at 18 months with cognitive, language, and pre-academic scores at 4–6 years. We are not aware of any previous studies reporting the predictive validity of the KDI, PSED, A not B task, or FCI. While we expected that correlations within domains would be stronger than across domains, we only found this pattern for socioemotional and home environment domains. Correlations between 18-month KDI motor scores and preschool fine motor scores were lower than those between the KDI and preschool visual search speed and pre-academic scores. The correlation of 18-month CDI vocabulary with preschool language score was about the same as those between the CDI and preschool pre-academic and cognitive factor scores. This suggests that performance on these tests at age 18 months may depend on the development of general cognitive abilities, more than specific motor and language skills.

The higher percentage of missing data on the direct assessments (5–9%) versus parent-report tools (< 0.5%) suggests that, at least at age 18–24 months, it is more common for children to refuse to perform activities during a direct assessment than for caregivers to respond that they do not know about their children’s abilities. The finding that excluding children who refused to perform some activities resulted in higher validity correlations than including them implies that such refusal decreases the accuracy of the scores on direct assessment tests, and parent-report tools may be preferable at this age.

Strengths of the study were the variety of assessment methods employed, the large number of children assessed in Ghana at 18 months and 4–6 years, and the collection of naturalistic speech samples for validation of the vocabulary checklist in Malawi. Another important strength was that we developed the items for the CDI vocabulary checklists through formative research in the local languages, rather than translating the items from English, which has been shown to result in item bias [32, 33]. A limitation of the study was that every tool was susceptible to measurement error, including NDW, which could be considered a gold standard measure. However, some caregivers may have encouraged their children to talk more than normal due to the recording, which would introduce measurement error. Therefore, where low correlations were found for these scores, it is difficult to determine which tool was performing poorly. For the DMC-II and MDAT, the data collectors had much less practice administering these tools compared to the other tests, which may partly account for the low correlations of these scores with NDW. In addition, the DMC-II and MDAT items capture a broad range of language skills beyond expressive vocabulary, which may also partly account for the lower correlations with NDW.

Despite these limitations, high correlations between concurrent measures provided evidence that both tools measured the same construct, while significant correlations between early and later measures provided evidence that the early score was a meaningful predictor of a child’s future performance. Another limitation was the small samples for evaluating concurrent validity of the language scores in Malawi. Although these sample sizes were powered to detect at least moderate validity correlations of 0.5, the samples were not randomly selected and may not be representative of the population. However, the pattern of relatively higher validity of the CDI compared to other tests was robust across samples and contexts.

## Conclusion

We have demonstrated the validity of a method to develop vocabulary checklists in new languages, based on the MacArthur-Bates CDI. This method meets many of the criteria that are desirable when selecting a test for use in a low- or middle-income country, including the following. Using this method, vocabulary checklists can be developed for a new language using a feasible adaptation process that takes about 2 weeks. The resulting vocabulary checklist can be administered relatively quickly (10 min) by personnel with no previous experience in developmental assessment. The method is appropriate for use in multilingual contexts. The vocabulary checklist scores reflect children’s current language ability, as demonstrated by correlations with other concurrent measures of language development, and predict children’s future language and cognitive ability. This is a promising method to assess early language development, which is an important skill that develops during early childhood and prepares children for success and pre-school and primary school.

## Additional file

Additional file 1: **Table S1.** Preschool Developmental Assessment Methods in Ghana. **Table S2.** Maternal and Household Characteristics Descriptive Statistics for Developmental Scores. (DOCX 38 kb)

## Abbreviations

BITSEA: Brief Infant/Toddler Social Emotional Assessment
CDI: Communicative Development Inventories
CHAT: Codes for the Human Analysis of Transcripts
CLAN: Computerized Language Analysis
DMC: Developmental Milestones Checklist
ECD: Early child development
FCI: Family care indicators
HOME: Home observation for the measurement of the environment
iLiNS: International Lipid-Based Nutrient Supplements
KDI: Kilifi Developmental Inventory
MDAT: Malawi Developmental Assessment Tool
NDW: Number of different words
PSED: Profile of Socioemotional Development
SDQ: Strengths and Difficulties Questionnaire
