# Supplementary material for: A method to develop vocabulary checklists in new languages and their validity to assess early language development
Source: J Health Popul Nutr. 2018 May 11;37:13. doi: 10.1186/s41043-018-0145-1 (PMC5948920; doi:10.1186/s41043-018-0145-1)
Supplement: Supplementary file 1 — Table S1. Preschool Developmental Assessment Methods in Ghana. Table S2. Maternal and Household Characteristics Descriptive Statistics for Developmental Scores. (DOCX 38 kb) [file 41043_2018_145_MOESM1_ESM.docx]

Online Supplemental Material for the Manuscript “A Method to Develop Vocabulary Checklists in New Languages and Their Validity to Assess Early Language Development”

Additional file 1

*Participants and Procedure: Predictive Validity of 18-Month Developmental Assessments*

The iLiNS-DYAD-G trial in Ghana was conducted in the semi-urban Somanya-Kpong area, in the Yilo Krobo and Lower Manya Krobo districts. Participants were visited at their last known address and recruited to the follow-up study. After providing informed consent, they were scheduled for a clinic visit for developmental assessment. Project staff also conducted an additional home visit to administer the Strengths and Difficulties Questionnaire and Home Observation for the Measurement of the Environment (HOME) Inventory.

*Preschool Assessments*

Language ability was assessed by the Body part naming and identification and Comprehension of instructions subtests of the Developmental Neuropsychological Assessment (NEPSY II) [1]. Pre-academic skills were assessed using the Parent’s Evaluation of Developmental Status (PEDS) developmental milestones test pre-academic subscale [2]. We assessed executive function using the head-toe, delay of gratification, and visual search tasks. The head-toe task was drawn from the International Development and Early Learning Assessment (IDELA) [3], the delay of gratification from Noble and colleagues [4], and the visual search task from the Supplementation with Multiple Micronutrients Intervention Trial (SUMMIT) [5]. We assessed visuospatial ability using a block design test based on the British Ability Scales (BAS II) pattern construction subtest [6]. We assessed declarative memory using a paired associate memory task from Baddeley and colleagues [7]. Motor function was assessed by the NIH Toolbox 9-hole pegboard test [8]. We assessed socio-emotional competence by caregiver interview using the Strengths and Difficulties Questionnaire (SDQ) [9], which is a widely-used assessment of socioemotional problems and strengths for young children (3-16 year old), and by direct observation using the Behavior Rating Scale adapted from The School Transition and Readiness (STAR) study (Obradovic & Yousafzai, personal communication), based on the Preschool Self-Regulation Assessment (PSRA) data collector report [10].

*Calculation of Preschool Z-Scores*

We calculated each preschool *z*-score, calculated by three-month age bands. Standard norming guidelines state that a sufficient sample size is 75 – 200 per age group. Age bands are expected to be smaller in the first year of age (1 month) and larger at later ages (2-3 months for toddlers and 6 months or 1 year for school children) [11]. Using 3-month age bands, our sample had about 100 per age group.

References

1. Korkman M, Kirk U, Kemp S: *NEPSY-II: A developmental neurospychological assessment.* San Antonio, TX: Pearson, Inc; 2007.

2. Brothers KB, Glascoe FP, Robertshaw NS: **PEDS: developmental milestones--an accurate brief tool for surveillance and screening.** *Clin Pediatr (Phila)* 2008, **47:**271-279.

3. Save the Children Foundation: **International Development and Early Learning Assessment (IDELA).** 2014.

4. Noble KG, Norman MF, Farah MJ: **Neurocognitive correlates of socioeconomic status in kindergarten children.** *Developmental Science* 2005, **8:**74-87.

5. Prado EL, Alcock KJ, Muadz H, Ullman MT, Shankar AH: **Maternal multiple micronutrient supplements and child cognition: a randomized trial in Indonesia.** *Pediatrics* 2012, **130:**e536-546.

6. Elliott CD: *British Ability Scales: Second Edition.* London: NFER-Nelson Publishing Co., Ltd; 1996.

7. Baddeley A, Gardner JM, Grantham-McGregor S: **Cross-cultural cognition: Developing tests for developing countries.** *Applied Cognitive Psychology* 1995, **9:**S173-S195.

8. Gershon RC, Cella D, Fox NA, Havlik RJ, Hendrie HC, Wagster MV: **Assessment of neurological and behavioural function: the NIH Toolbox.** *The Lancet Neurology* 2010, **9:**138-139.

9. Goodman R: **The Strengths and Difficulties Questionnaire: a research note.** *Journal of child psychology and psychiatry, and allied disciplines* 1997, **38:**581-586.

10. Smith-Donald R, Raver CC, Hayes T, Richardson B: **Preliminary construct and concurrent validity of the Preschool Self-Regulation Assessment (PSRA) for field-based research.** *Early Childhood Research Quarterly* 2007, **22:**173-187.

11. Glascoe FP, Marks KP, Macias MM, Howard B, Sturner R, Aydin M, Zahnd E, Grant D, Holtby S, Woods SK: **Test Construction and Psychometrics, Quality Improvement and Other Research in Developmental-Behavioral Screening.** In *Identifying and addressing developmental-behavioral problems: A Practical Guide for Medical and Non-Medical Professionals, Trainees, Researchers and Advocates.* Edited by Glascoe FP, Marks KP, Poon JK, Macias MM: PEDStest.com, LLC; 2013: 424-452

*Table S1*. Preschool Developmental Assessment Methods in Ghana

| Preschool Score | Method |
| --- | --- |
| Cognitive |  |
| Pre-Academic Skills [2] | We administered an adapted version of the Parents’ Evaluation of Developmental Status: Developmental Milestones pre-academic subscale by direct administration to the child. We calculated the total score as the sum of the item scores, then calculated z-scores by 3-month age bands. |
| Paired-Associate Memory [7] | In this test, the child is shown a page printed with pictures of eight objects and told novel names for each object. The tester then says the novel name of the object and asks the child to point to the correct picture. The learning score was the sum of the number correct on eight learning trials. The delayed recall score was the sum of the number correct on two trials administered after a delay of median 7 minutes (IQR 6-11). For each score, we calculated the z-score by 3-month age band then calculated the child’s average z-score. |
| Language [1] | We administered the Developmental Neuropsychological Assessment (NEPSY-II) body part naming and identification sub-tests and comprehension of instructions sub-test. For each of the three scores, which represented the total correct on that sub-test, we calculated the z-score by 3-month age band then calculated the child’s average z-score. |
| Block Design [5] | We administered a block design test, based on the British Ability Scales [6] Pattern Construction sub-test previously adapted in Indonesia. In this test, the child was asked to copy increasingly complex patterns with wooden blocks. We calculated the total correct, then calculated z-scores by 3-month age bands. |
| Visual Search [5] | This test was also based on a previous adaptation of the NEPSY visual search sub-test in Indonesia. The child was presented with an array of pictures and asked to mark the pictures that matched the target picture as quickly as possible. The time per correct target was calculated for each of two trials. We calculated z-scores by 3-month age band then calculated the child’s average z-score. |
| Inhibitory Control: Head/Toe task [3] | We assessed inhibitory control using the head/toe subtest of the International Development and Early Learning Assessment (IDELA). Children were first instructed to touch their head or toes in accordance with the assessor’s verbal cue. In the test trials, children were instructed to do the opposite of the verbal cue (e.g., touch their head when the assessor said “toes”). The score was the sum of 5 item scores, each scored 0-2. We calculated z-scores by 3-month age band. |
| Inhibitory Control:  Delay of Gratification [4] | After the first test was completed, the child was shown a variety of colorful treats. The child was given the choice of having either one treat immediately or of having more treats later, specifically two, three or four treats at the end of the second, third and fourth tasks, respectively. The score was the number of treats the child waited to receive. |
| Cognitive Factor Score | We calculated an overall cognitive factor score as the first factor of a factor analysis using the principal-axis factoring method including all cognitive z-scores except the delay of gratification score, which was the only score that was not strongly associated with the other scores. This component accounted for 79% of the variance in these scores. |
| Motor |  |
| Fine Motor [8] | In the National Institute of Health (NIH) Toolbox 9-Hole Pegboard test, the score is the number of seconds for the child to place and remove 9 pegs in a pegboard, first with the dominant hand then the non-dominant hand. For each score, we calculated the z-score by 3-month age band then calculated the child’s average z-score. |
| Socioemotional |  |
| Strengths and Difficulties Questionnaire [9] | In an interview, the child’s caregiver rated 25 items concerning the child’s behavior on a scale from 0-2. We calculated the total difficulties score (20 items, including emotional symptoms, conduct problems, hyperactivity/inattention, and peer relationship problems) and prosocial score (5 items), then calculated z-scores by 3-month age bands. |
| Behavior Rating Scale | The Behavior Rating Scale was adapted from the Preschool Self-Regulation Assessment (PSRA) Assessor Report [10] in a previous study in Pakistan (Obradovic & Yousafzai, personal communication). The assessor observed the child during the test session and rated the child’s behavior on 10 items, each scored 0-3, such that a higher score indicated better behavior (e.g., child waits patiently for new tasks to begin; child does not show frequent feelings of sadness/anger). |

*Table S2*. Maternal and Household Characteristics Descriptive Statistics for Developmental Scores

|  | Language Validation Study | | iLiNS-DOSE | | iLiNS-DYAD-M | | iLiNS-DYAD-G | |
| --- | --- | --- | --- | --- | --- | --- | --- | --- |
|  | *n* | *mean (SD)* | *n* | *mean (SD)* | *n* | *mean (SD)* | *n* | *mean (SD)* |
| Maternal education (y) | 29 | 5 (4) | 1358 | 5 (4) | 671 | 4 (3) | 1023 | 8 (4) |
| Maternal Age (y) |  |  | 1358 | 26 (6) | 675 | 25 (6) | 1023 | 27 (5) |
| Maternal Body Mass Index (kg/m^2^) |  |  | 1379 | 22 (3) | 671 | 22 (3) | 1006 | 25 (5) |
| Household used unimproved water source^1^: n (%) |  |  | 1200 | 92 (8%) | 674 | 60 (9%) | 1021 | 67 (7%) |
| Household used unimproved toilet facility^2^: n (%) |  |  | 1200 | 1176 (98%) | 674 | 610 (91%) | 1021 | 26 (3%) |
| CDI Vocabulary | 30 | 47 (28) | 1382 | 32 (23) | 675 | 30 (23) | 1022 | 31 (19) |
| KDI Motor |  |  | 1338 | 38 (4) | 670 | 38 (4) | 918 | 39 (3) |
| PSED social competence |  |  | 1385 | 7 (3) | 675 | 7 (3) | 1022 | 7 (2) |
| PSED behavior problems |  |  | 1385 | 7 (3) | 675 | 8 (3) | 1022 | 8 (2) |
| PSED total score |  |  | 1381 | 15 (5) | 674 | 15 (5) | 1022 | 15 (4) |
| DMC-II Language | 30 | 21 (4) |  |  |  |  |  |  |
| MDAT Language | 30 | 16 (4) |  |  |  |  |  |  |
| A not B Total Correct |  |  |  |  |  |  | 880 | 6 (2) |
| FCI variety of play materials |  |  |  |  |  |  | 1023 | 3 (1) |
| FCI activities with caregivers |  |  |  |  |  |  | 1023 | 5 (3) |
| FCI total score |  |  |  |  |  |  | 1023 | 8 (3) |
| Pre-academic score |  |  |  |  |  |  | 958 | 18 (6) |
| Paired associate learning score |  |  |  |  |  |  | 958 | 21 (5) |
| Body part naming |  |  |  |  |  |  | 960 | 5(2) |
| Body part identification |  |  |  |  |  |  | 960 | 8 (2) |
| Comprehension of instructions |  |  |  |  |  |  | 962 | 9 (3) |
| Block design score |  |  |  |  |  |  | 960 | 17 (6) |
| Visual search speed |  |  |  |  |  |  | 958 | 5 (2) |
| Head/Toe task score |  |  |  |  |  |  | 958 | 4 (4) |
| Delay of gratification score |  |  |  |  |  |  | 960 | 2 (1) |
| Pegboard dominant hand |  |  |  |  |  |  | 963 | 31 (5) |
| Pegboard non-dominant hand |  |  |  |  |  |  | 959 | 37 (6) |
| Socioemotional competence |  |  |  |  |  |  | 959 | 8 (2) |
| Socioemotional difficulties |  |  |  |  |  |  | 959 | 13 (4) |
| Behavior rating scale |  |  |  |  |  |  | 962 | 24 (4) |
| HOME Inventory |  |  |  |  |  |  | 928 | 28 (5) |

^1^Unimproved water was defined as unprotected well or surface water (well, pond, lake, etc); Improved water was defined as indoor or outer plumbing or pipe, water vendor, borehole, or protected well.

^2^Unimproved toilet was defined as a regular pit latrine, pan/bucket, or no toilet facility; Improved toilet was defined as an improved pit latrine, water closet, or flush toilet
